# Addressing the Global Challenge of Nitrous Oxide Misuse Through a Multidisciplinary Approach: Example of the PROTOSIDE Network

**DOI:** 10.3390/toxics13060466

**Published:** 2025-05-31

**Authors:** Guillaume Grzych, Remy Diesnis, Thierry Dupré, Jean Paul Niguet, Emeline Gernez, Damien Denimal, Sylvie Deheul, Jean Claude Guichard, Damien Scliffet, Bruno Mégarbane, Isabelle Redonnet-Vernhet, Alexandra Boucher, Anas Bennis, Laurent Karila, Etienne Cavalier, Benjamin Rolland, Christophe Riou, Cécile Bossaert, Anthony Chauvin

**Affiliations:** 1 CHU Lille, Service Biochimie Automatisée-Protéines, 59000 Lille, France; 2 Emergency Departement, CH Roubaix, 59100 Roubaix, France; remy.diesnis@ch-roubaix.fr; 3 Département de Biochimie, AP-HP, Hôpital Bichat, Biochimie, 75018 Paris, France; thierry.dupre@aphp.fr; 4 Service de Neurologie, Hôpital Saint Vincent de Paul-GHICL, 59000 Lille, France; niguet.jeanpaul@ghicl.net; 5 CHU de Lille, Centre de Biologie Pathologie Génétique, 59000 Lille, France; emeline.gernez@chu-lille.fr; 6 CHU Dijon Bourgogne, INSERM U1231, Université Bourgogne Europe, 21000 Dijon, France; damien.denimal@chu-dijon.fr; 7 Inserm, UMR S1171, CHU Lille, Service de Pharmacologie Médicale, Université de Lille, 59000 Lille, France; sylvie.deheul@chu-lille.fr; 8 CEIP-Addictovigilance, Service de Pharmacologie Médicale, CHRU de Lille, 59045 Lille, France; 9 Unité Sanitaire en Milieu Pénitentiaire USMP, 59000 Lille, France; jean-claude.guichard@chu-lille.fr; 10 CH de Lens, Addictologie, 62300 Lens, France; dscliffet@ch-lens.fr; 11 Réanimation Médicale et Toxicologique, Hôpital Lariboisière, Université Paris Cité, 75013 Paris, France; bruno.megarbane@aphp.fr; 12 Biochimie, CHU Bordeaux, INSERM U1211 MRGM, Université Bordeaux, 33076 Bordeaux, France; isabelle.redonnet-vernhet@chu-bordeaux.fr; 13 Centre d’Addictovigilance, Hospices Civils de Lyon, 69000 Lyon, France; alexandra.boucher@chu-lyon.fr; 14 Assistance Publique-Hôpitaux de Paris, Service de Neurologie, Groupe Hospitalier Universitaire Paris Sud, Hôpital Bicêtre, 94270 Le Kremlin-Bicêtre, France; bennisanas123@gmail.com; 15 Centre d’Enseignement, de Recherche et de Traitement des Addictions, Hôpital Universitaire Paul-Brousse (AP-HP), Université Paris-Saclay, 94804 Villejuif, France; laurent.karila@aphp.fr; 16 Department of Clinical Chemistry, CIRM, University of Liege, 4000 Liège, Belgium; etienne.cavalier@chuliege.be; 17 Service Universitaire d’Addictologie de Lyon (SUAL), Hospices Civils de Lyon, CH Le Vinatier, 69002 Lyon, France; benjamin.rolland@chu-lyon.fr (B.R.); christophe.riou@chu-lyon.fr (C.R.); 18 PSYR, CRNL, INSERM U1028, CNRS UMR5292, UCBL1, 69500 Lyon, France; 19 CHU de Lille, Service des Urgences Adultes, SAMU, 59000 Lille, France; cecile.bossaert@chu-lille.fr; 20 Emergency Department, Hôpital Lariboisière, APHP, 75010 Paris, France; anthony.chauvin@aphp.fr

**Keywords:** nitrous oxide, addiction, neurology, toxicology, international network, multidisciplinary, methylmalonic acid, homocysteine, biomarkers

## Abstract

Nitrous oxide (N_2_O) was originally used for medical and industrial purposes, but its recreational use has dramatically increased, raising a major global public health concern. Chronic inhalation is associated with neurological, metabolic, and psychiatric complications, as well as addiction. To address these challenges, the PROTOSIDE network was developed to provide a multidisciplinary approach to management and prevention. This initiative relies on competence centers integrating specialists in emergency medicine, neurology, clinical biochemistry, and addiction medicine. PROTOSIDE aims to standardize diagnostic protocols, optimize patient care pathways, and strengthen addictovigilance. A strong emphasis is placed on prevention, including awareness campaigns and collaboration with healthcare professionals and educators. By facilitating access to advanced biochemical analyses (homocysteine, methylmalonic acid) and promoting international guidelines, PROTOSIDE represents an innovative model for a global response to N_2_O misuse. This integrated approach enhances clinical management, reduces complications, and harmonizes public health strategies.

## 1. Context, State of the Art, and Challenges

The increasing recreational use of nitrous oxide (N_2_O), originally developed for medical and industrial purposes, has recently become a major global public health concern [1,2]. Scientific studies highlight the severe harms resulting from recreational N_2_O use, including neurological, metabolic, and psychiatric complications, as well as addiction [3].

### 1.1. A Historical Shift: From Medical Use to Recreational Misuse

**Origins of medical use and early recreational consumption**

Nitrous oxide (N_2_O) was first identified in the late 18th century by Joseph Priestley and later popularized by Sir Humphry Davy, who experimented with its psychoactive properties in 1799. Davy’s observations of euphoria and dissociation led to its adoption as an anesthetic agent in dentistry and surgery by the mid-19th century. The substance quickly became a cornerstone of modern anesthetic practice, favored for its rapid onset, short duration of action, and minimal residual effects [4].

However, recreational misuse of N_2_O is not a new phenomenon. Historical accounts suggest that early anesthetic demonstrations in the 19th century often turned into public spectacles, where attendees inhaled the gas for entertainment, leading to fits of laughter—hence its nickname, “laughing gas”. By the early 20th century, sporadic cases of misuse among medical professionals and industrial workers were documented, particularly in settings where the gas was readily available [1].

Despite its long history in medicine, N_2_O was never classified as a controlled substance in most jurisdictions due to its legitimate medical applications and relatively low potential for acute toxicity. This regulatory leniency, combined with technological advances in gas storage and distribution, has played a pivotal role in facilitating its transition from clinical to recreational settings [2].

**The shift to mass recreational use**

**Figure 1:**
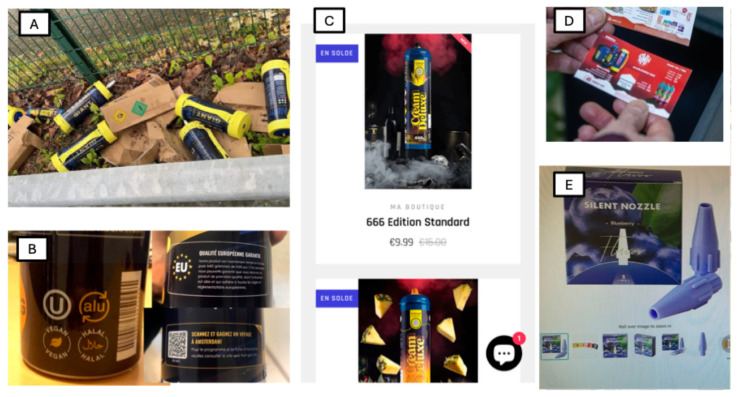
Extensive Distribution and Aggressive Marketing Strategies. (**A**) Current forms of nitrous oxide consumption: large cylinders containing the equivalent of several hundred small cartridges. (**B**) Marketing labels on nitrous oxide canisters: “Vegan,” “Halal,” “European Quality,” and a promotional game offering a trip to Amsterdam. (**C**) Sale and aggressive marketing of nitrous oxide via social media platforms. (**D**) Leaflet advertising for nitrous oxide sales, distributed in mailboxes. (**E**) Canister adapter designed to add flavor to nitrous oxide.

Although isolated cases of N_2_O abuse had been reported throughout the 20th century, widespread recreational consumption surged in the 21st century, fueled by several key factors (Figure 1).

One of the primary drivers is the mass commercial availability. N_2_O is legally sold for culinary and industrial purposes, particularly in the form of pressurized cartridges used in whipped cream dispensers [5]. The expansion of online marketplaces and non-traditional retail distributors has dramatically increased access [6].

In parallel, social media platforms such as TikTok, Instagram, and YouTube have glamorized N_2_O consumption, showcasing its effects while downplaying the potential dangers [7]. These platforms often showcase content featuring “balloon challenges” or binge-like consumption patterns at parties and music festivals [8].

Another key factor lies in the misconception of safety surrounding N_2_O. Compared to other inhalants (e.g., volatile solvents, nitrites, gasoline), N_2_O is perceived as “harmless” due to its medical applications and absence of strong withdrawal symptoms [9]. In addition, many users incorrectly assume that its rapid elimination from the body prevents long-term harm, unaware of the biochemical impact on vitamin B12 metabolism [10].

Compounding the issue is a notable shift toward high-dose and chronic use. Early recreational use primarily involved small “whippet” cartridges, limiting the amount of gas that could be consumed at one time [1]. However, the rise of large industrial-grade N_2_O canisters (containing several liters of gas) has led to prolonged sessions and higher cumulative exposure [1]. This transition is particularly concerning as chronic, high-dose use significantly increases the risk of neurological complications, including myelopathy and peripheral neuropathy [11].

**The role of unregulated distribution channels**

One of the most pressing challenges in mitigating N_2_O misuse is the unregulated nature of its distribution, which occupies a legal gray zone. Unlike alcohol or tobacco, it is widely available for legitimate industrial, medical, and culinary purposes, yet its recreational use is rarely addressed in legislation. Online retailers and international suppliers have exploited legal loopholes, making bulk purchases easier than ever, and there is limited enforcement of existing regulations, particularly in countries where recreational use is not explicitly prohibited. This lack of oversight has fueled an underground market where users can purchase large quantities without restriction, contributing to the rapid normalization of recreational N_2_O consumption.

### 1.2. The Epidemiological Surge: A Growing Global Concern

#### 1.2.1. Europe: A Hub for Nitrous Oxide Consumption

Over the past decade, Europe has emerged as a central hotspot for N_2_O misuse, with alarming trends reported across multiple countries. The European Monitoring Centre for Drugs and Drug Addiction (EMCDDA) has identified N_2_O as the most widely used psychoactive substance among young adults [2,5].

According to the Global Drug Survey 2021, nitrous oxide ranked as the 14th most commonly used recreational substance globally. Its use is especially prevalent among adolescents and young adults under the age of 25, with studies cited in the 2023 WHO report estimating past-year prevalence between 4% and 15% in countries such as Denmark, the Netherlands, and the UK. In France, over 134 cases of nitrous-oxide-related neurological complications were reported to poison control centers in 2020 alone, and hospital admissions for N_2_O toxicity continue to rise across Europe [2,12].

This widespread use is likely driven by several factors: the legal status of N_2_O in many countries, its accessibility through whipped cream chargers and larger canisters, low cost, and its misleading reputation as a “safe” or “legal” high. Notably, the shift from recreational use of small whipped cream cartridges to high-volume canisters and tanks has dramatically increased the risk of severe toxic effects, including irreversible neurological damage and even death. Despite its trivialized image, N_2_O poses real and escalating dangers that warrant urgent and coordinated public health responses.

**Key epidemiological findings according to the EMCDDA report**

According to the latest report from the EMCDDA, several key epidemiological trends have emerged in recent years regarding nitrous oxide misuse.

One of the most alarming findings is a five-fold increase in emergency room admissions linked to N_2_O-related complications. This sharp rise underscores a growing public health concern that spans across multiple European countries.

Among the nations most affected, the Netherlands, France, and the United Kingdom report the highest prevalence rates of recreational N_2_O use [12]. This pattern is particularly pronounced in urban areas where nightlife culture thrives. Indeed, the clustering of N_2_O misuse is frequently observed in environments such as music festivals, nightclubs, and university parties, where peer influence and easy access converge to drive consumption.

Compounding this issue is a notable shift in the method of use. Whereas users once relied on small whipped cream cartridges, the market has seen a transition toward large industrial-sized canisters. These high-volume containers enable prolonged inhalation sessions, significantly increasing both the intensity and duration of intoxication. This change in usage patterns has been directly associated with a rise in severe neurological outcomes, marking a dangerous evolution in the recreational use of nitrous oxide.

**Regulatory responses and effectiveness**

In response to the growing misuse of nitrous oxide, some European countries have implemented regulatory measures aimed at curbing its recreational use. The Netherlands was among the first to act decisively, banning sales of N_2_O for recreational purposes in January 2023. Despite this legislative move, illegal distribution networks have quickly adapted, and unregulated markets continue to thrive [13].

France has taken a more moderated approach, introducing age restrictions and setting limits on the quantity of N_2_O that can be purchased. However, these measures suffer from inconsistent enforcement, diminishing their overall impact.

Similarly, the United Kingdom reclassified nitrous oxide as a Class C drug in 2023, effectively criminalizing its recreational use. Yet, early evidence indicates that illicit sales remain readily accessible through online platforms, underscoring the limitations of prohibition-based strategies.

Preliminary data suggest that such restrictions have not led to a substantial decline in use. This reinforces the urgent need for complementary harm reduction policies, including education, awareness campaigns, and community-based interventions, to address the multifaceted nature of N_2_O misuse.

#### 1.2.2. Emerging Concerns: From Acute to Chronic Health Consequences

Historically, most N_2_O-related medical cases involved acute intoxication, presenting as transient dizziness, nausea, or mild euphoria. However, recent clinical reports suggest a shift toward severe, long-term complications [14]. Increasingly, clinicians are reporting chronic neurological syndromes associated with cumulative exposure [11]. Notably, a growing number of cases involve irreversible nerve damage in daily users [11]. These outcomes are often exacerbated by delayed diagnosis and mismanagement, as symptoms may be nonspecific and unfamiliar to many healthcare providers, leading to progressive disability [15,16].

These trends underscore the urgent need for enhanced surveillance, clinical awareness, and standardized treatment guidelines.

### 1.3. Clinical and Biological Consequences of Nitrous Oxide Misuse [1]

#### 1.3.1. Neurological Manifestations: Myeloneuropathy and Central Nervous System Impairment

One of the most alarming consequences of chronic N_2_O exposure is its profound impact on the nervous system, leading to peripheral neuropathy and myelopathy [17]. The mechanism of action involves an irreversible oxidation of vitamin B12 (cobalamin), leading to a functional deficiency that disrupts key metabolic pathways [1]. This process also causes demyelination of the spinal cord (subacute combined degeneration, SCD), leading to motor dysfunction, paresthesia, and proprioceptive ataxia [18].

**Clinical presentations**

The impact of N_2_O misuse on the nervous system can lead to myelopathies, characterized by spinal cord degeneration, affecting vibration sense, coordination, and motor function [19], as well as peripheral neuropathies, responsible for paresthesia, numbness, muscle weakness, and loss of deep tendon reflexes [20].

Cognitive and psychiatric impairment may also occur, including memory loss, mood disturbances, anxiety, and, in severe cases, psychotic symptoms [21,22].

Electrophysiological findings often reveal prolonged motor and sensory conduction velocities, reflecting axonal damage [20].

#### 1.3.2. Biochemical and Metabolic Disruptions: Methylmalonic Acid and Homocysteine Dysregulation

The major biochemical disruption caused by N_2_O misuse is the functional inactivation of vitamin B12, also called cobalamin [23,24,25]. Vitamin B12 is a crucial cofactor for two essential enzymes: methionine synthase (MS) and methylmalonyl-CoA mutase (MM-CoAM). Thus, this oxidation disrupts both the methylation and mitochondrial metabolic cycles:

- Methionine synthase, which requires methylcobalamin as a cofactor, catalyzes the remethylation of homocysteine into methionine. When MS activity is blocked, homocysteine accumulates in plasma—a biochemical hallmark of impaired one-carbon metabolism. This elevation is clinically relevant due to its association with vascular and neurological complications.
- Methylmalonyl-CoA mutase, on the other hand, depends on adenosylcobalamin (another active form of vitamin B12) and facilitates the conversion of methylmalonyl-CoA to succinyl-CoA in mitochondria. Inactivation of this enzyme leads to accumulation of methylmalonic acid (MMA), a marker of intracellular B12 dysfunction.

**As a consequence, nitrous oxide intoxication can lead to (Table 1):**

- Increased plasma homocysteine (marker of impaired methylation cycles) [26,27].
- Accumulation of methylmalonic acid (MMA) [10,28,29].
- Reduction in methionine synthesis, affecting myelin integrity [30].

**Table 1:** Key biochemical markers in N_2_O toxicity.

Biological Markers	Pathophysiological Significance
Plasma homocysteine	Sensitive marker of N_2_O exposure; reflects impaired methionine metabolism [10].
Methylmalonic acid (MMA)	More specific to B12-related neurological dysfunction; correlated with clinical severity [10].
Methionine levels	Low methionine is associated with myelin damage, linked to neurological outcomes [30].

**Key Takeaways:**

- Homocysteine rises rapidly post-exposure, returning to normal within days [1,31].
- MMA remains elevated longer and correlates better with clinical severity [10,32,33].
- Vitamin B12 levels alone are unreliable, as they may appear normal despite severe toxicity [10].

#### 1.3.3. Hematological and Bone Marrow Effects

Chronic N_2_O exposure could in theory also mimic megaloblastic anemia [34] due to its impact on vitamin B12 related to DNA synthesis and erythropoiesis; however, studies have not reported this effect yet [35].

#### 1.3.4. Cardiovascular and Thromboembolic Complications

Recent research has demonstrated that N_2_O misuse increases thrombotic risk, primarily via hyperhomocysteinemia-induced endothelial dysfunction [36,37]. The literature reports cerebral venous thrombosis (CVT) [38]; myocardial infarction and deep vein thrombosis (DVT), likely due to homocysteine-related vascular inflammation and endothelial dysfunction [39]; and ischemic strokes associated with elevated homocysteine levels and reduced nitric oxide bioavailability, leading to prothrombotic states [40].

**Figure 2:**
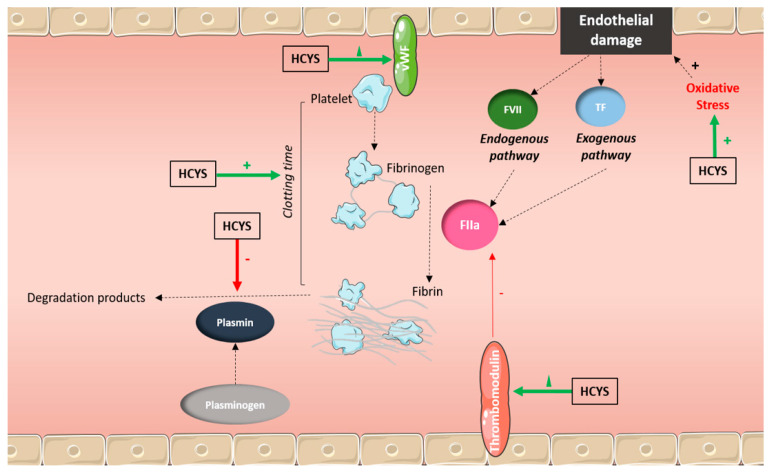
Possible mechanisms of vascular injury and cardiovascular events related to high homocysteine (HCYS) caused by nitrous oxide (N_2_O) abuse. Endothelial damages: Homocysteine promotes oxidative stress, damaging blood vessels. Pro-coagulant effects: Elevated homocysteine activates platelets and coagulation cascades.

The hypothetical mechanisms of vascular injury caused by N_2_O misuse are multiple (Figure 2). First, the hyperhomocystenemia caused by consumption can lead to endothelial dysfunction as homocysteine promotes oxidative stress, thus damaging blood vessels [41,42,43]. Moreover, high levels of homocysteine are responsible for pro-coagulant effects via the activation of platelets and coagulation cascades [44]. Finally, impaired NO signaling leads to reduced vasodilation and increased blood pressure [45].

#### 1.3.5. Psychiatric and Cognitive Consequences

Beyond its neurometabolic toxicity, N_2_O misuse is associated with psychiatric disorders, including anxiety and depressive symptoms (reported in chronic users) [46]; psychotic episodes and hallucinations, especially in those with underlying psychiatric conditions [22]; and memory impairments and executive dysfunction, possibly due to N_2_O-induced oxidative stress and impaired methylation pathways [47].

Some case studies suggest that N_2_O dependence and withdrawal symptoms may develop, particularly in high-frequency users, although clear diagnostic criteria for N_2_O addiction remain debated.

To address these growing challenges, the PROTOSIDE network (https://protoside.com/en/) was developed to formulate proposals for a coordinated, multidisciplinary, and global response.

### 1.4. Road Safety and Neurocognitive Risks of Nitrous Oxide Use

Recreational use of nitrous oxide (N_2_O) not only presents neurological and metabolic risks but also poses a significant emerging threat to road safety. Although its euphoric effects are short-lived, its impact on cognitive and motor functions may last well beyond the acute phase, impairing driving performance and increasing accident risk [48].

Recent studies and accident reports across Europe—particularly in France, the Netherlands, and the UK—highlight the growing involvement of N_2_O in traffic incidents. For instance, the French National Road Safety Observatory (ONISR) estimates that up to 11% of severe accidents in 2023 involved psychoactive substances, including N_2_O. In the Netherlands, N_2_O-related road accidents rose by 80% between 2019 and 2021, underscoring the scale of the problem [48].

The mechanisms of impairment [48] are multiple. First, the short-term effects of N_2_O include dizziness and mental confusion, which can impair spatial orientation and attention. Then, mid- to long-term effects include loss of motor coordination, which affects precise control of vehicle handling. Slowed reaction times can also occur and compromise the driver’s ability to respond to sudden obstacles. Euphoria and overconfidence can lead to risk-taking behaviors, including speeding and reckless maneuvers. Polydrug use, especially with alcohol or cannabis, further exacerbates driving impairment.

Despite these risks, N_2_O remains difficult to detect in roadside testing due to its rapid elimination and the absence of standardized field detection tools. This creates a critical gap in enforcement and makes it difficult to sanction intoxicated drivers.

Current challenges in detection:

N_2_O is not detectable by standard alcohol or drug screening tools used in roadside tests. Indeed, the rapid absorption and elimination of this anesthetic gas complicates its detection in biological matrices, as N_2_O has a very short half-life of only a few minutes. Moreover, blood and urine analyses require advanced laboratory methods not available in field settings. Gas chromatography–mass spectrometry (GC-MS) is one of the available methods, but it faces several limitations, including difficulty in selecting an optimal internal standard, limited sensitivity, and potential risk of leaking during sampling, extraction, and analysis. Headspace-GC-MS appears promising but requires further validation for routine laboratory use. Infrared spectroscopy techniques offer high sensitivity for measuring N_2_O in air but are not applicable to biological matrices. Neither of these sophisticated methods are conducive to use roadside and by non-laboratorians.

Given these challenges, indirect markers reflecting the physiological impact of N_2_O exposure may need to be explored for reliable detection in forensic and clinical settings. However, indirect markers (homocysteine, methylmalonic acid) are not specific enough for acute detection.

Finally, there is no established legal limit to define N_2_O intoxication relevant for driving regulation.

Public health implications:

Given the prevalence of use among adolescents and young adults and its rising association with road traffic fatalities, road safety must be a key target of public prevention campaigns. The PROTOSIDE network integrates this dimension by including road safety awareness in public educational efforts; collaborating with law enforcement and forensic toxicology experts to develop detection protocols; and advocating for regulatory reforms to classify N_2_O driving under drug-impaired driving laws.

Further research is urgently needed to define psychomotor thresholds of impairment under N_2_O; validate point-of-care detection tools; and propose legal frameworks and sanctions.

## 2. Why a Global Network Was Needed

Medical literature has established a sharp rise in N_2_O recreational use worldwide, with areas all over the world reporting exponential increases in related complications (1). The complications associated with recreational N_2_O use include subacute combined degeneration of the spinal cord, vitamin B12 deficiency, and thromboembolic events. All these complications often require specialized care, which is not widely accessible or consensual (3).

Gaps in diagnosis protocols, management, and prevention strategies represent additional difficulties. Founding PROTOSIDE was driven by the need to offer guidance on clinical practices and protocols for diagnosing and managing N_2_O misuse (4); offer an integrated network for comprehensive patient care [49]; and raise awareness among healthcare professionals and the public on the dangers of recreational N_2_O use [50].

## 3. Multidisciplinary Competence Centers: The Foundation of PROTOSIDE

**Figure 3:**
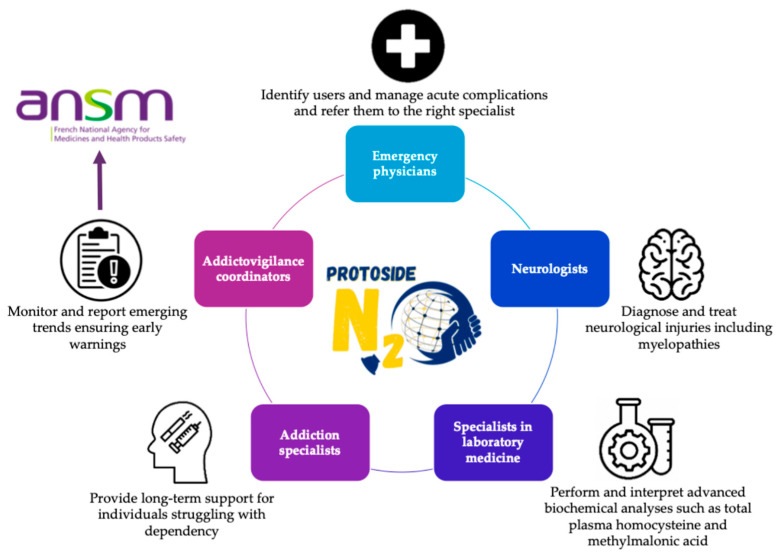
Multidisciplinary approach and team for patients with nitrous oxide care related to PROTOSIDE (www.protoside.com/en/) network competence centers; integration of expertise from various sources to ensure comprehensive care.

The strength of PROTOSIDE (Platforms and Resources for Orientation, Treatment, and Organization of Solutions for Intoxications with N_2_O, Diagnostics, and Education) lies in its network of competence centers, which integrate various expertise to ensure comprehensive care (5). Centers are required to include specialists such as (Figure 3):

- Emergency physicians, to identify users, manage acute complications, and refer them to the right specialists;
- Neurologists, to diagnose and treat neurological injuries, including myelopathies [51];
- Specialists in laboratory medicine, equipped to perform advanced biochemical analyses such as total plasma homocysteine and MMA concentration measurements (4,6);
- Addiction specialists, to provide long-term support for individuals struggling with dependency [52];
- Addictovigilance coordinators, to monitor and report emerging trends, ensuring early warnings.

This multidisciplinary structure ensures that all aspects of patient care are addressed, from acute treatment to rehabilitation.

## 4. Comprehensive Patient Care and Prevention

PROTOSIDE adopts a holistic approach to patient care, aiming to offer an integrated pathway that includes:

1. Early detection and diagnosis, leveraging multidisciplinary expertise and advanced analytical tools.
2. Management, addressing acute complications, metabolic impairments, and neurological damage.
3. Addiction support, including tailored programs to help patients overcome dependency on nitrous oxide.
4. Long-term follow-up and coordination with primary care to ensure continuity of care through collaboration with general practitioners and specialists.

In addition to clinical care, PROTOSIDE places significant emphasis on prevention and public awareness. Key initiatives include first educational campaigns targeting the public, particularly young people, to highlight the risks of recreational N_2_O use. There is also an important need for media outreach to inform communities based on articles, interviews, and social media about the health dangers associated with N_2_O misuse. Finally, collaboration with schools and community organizations is essential, as is engaging with educators and youth leaders to promote safe behaviors and discourage N_2_O misuse.

## 5. Knowledge Sharing and Global Collaboration

PROTOSIDE is also committed to advancing knowledge and harmonizing global practices using:

- Scientific conferences, bringing together experts to share research, discuss best practices, and align strategies to manage N_2_O misuse.
- Training programs for healthcare professionals, providing comprehensive modules on diagnosing, treating, and preventing N_2_O-related complications.
- Publications and research, contributing to the medical literature on mechanisms, health impacts, and management of N_2_O misuse.

## 6. A Global Model for Public Health Action

By combining multidisciplinary expertise, international collaboration, and a strong emphasis on public education, PROTOSIDE sets a benchmark for addressing complex public health challenges. The network provides a robust framework for tackling the growing problem of N_2_O misuse while fostering innovation in diagnostics, treatment, and prevention.

Healthcare professionals and organizations interested in joining this effort or accessing resources can visit the official PROTOSIDE website at https://protoside.com/en/.

## Data Availability

Data sharing is not applicable (only appropriate if no new data is generated or the article describes entirely theoretical research).
